# Functional Outcomes of the Management of Acromioclavicular Joint Dislocation With a Clavicle Hook Plate: A Retrospective Study

**DOI:** 10.7759/cureus.73898

**Published:** 2024-11-18

**Authors:** Muhammad Mannan, Asif Afridi, Khandaker T Ahmed, Shahzeen Eisha, Muhammad ishfaq Mazari

**Affiliations:** 1 Trauma and Orthopaedics, Sheikh Zayed Medical College and Hospital, Rahim Yar Khan, PAK; 2 Orthopaedic Surgery, University Hospital Birmingham, Birmingham, GBR; 3 Trauma and Orthopaedics, Hayatabad Medical Complex, Peshawar, PAK; 4 Trauma and Orthopaedics, Queen Elizabeth Hospital Birmingham, Birmingham, GBR; 5 Trauma and Orthopaedics, Birmingham Heartlands Hospital, Birmingham, GBR; 6 Trauma and Orthopaedics, Royal College of Surgeons in Ireland, Brighton, GBR; 7 Orthopaedic Surgery, Sheikh Zayed Medical College and Hospital, Rahim Yar Khan, PAK

**Keywords:** acromioclavicular joint, acute dislocation, constant murley score, functional outcome, hook plate

## Abstract

Background: Dislocation of the acromioclavicular joint (ACJ), accounting for 9%-12% of all shoulder injuries, is a frequent shoulder problem. Clavicular hook plates have proven to be a successful implant choice for surgical management. The benefit of this method is that it preserves the diarthrosis of the ACJ while reducing dislocation. Positive outcomes with this approach have been reported.

Objective: This study aimed to quantify functional recovery using the Constant Murley Score (CMS) in patients with ACJ dislocations treated with a clavicle hook plate.

Methodology: The retrospective study was conducted at the orthopedic department of Sheikh Zayed Medical College and Hospital, Rahim Yar Khan, Pakistan, for over one year, from January 20, 2021, to 19 January, 2022. A total of 40 individuals were identified who were operated on for acute ACJ dislocation with hook plates in the department and were included in the research. All patients underwent open reduction and internal fixation with a clavicular hook plate, which was usually removed three months after surgery. The functional outcome was evaluated using the CMS on the day before plate removal, three months after plate removal, and six months following surgery.

Results: Of the participants, 28 (70%) were male, and 12 (30%) were female. The mean CMS was 72.25 ± 3.95 (satisfactory) at 24 weeks and 90.45 ± 1.9 (excellent) eight weeks after the removal of the plate.

Conclusion: The hook plate is an excellent choice for treating acute ACJ dislocation. Radiographic and functional assessments indicate that these plates provide good outcomes.

## Introduction

Acromioclavicular joint (ACJ) dislocation is a common shoulder injury, making up 9%-12% of all shoulder injuries [1]. The severity of ACJ dislocation depends on the degree of displacement of the distal clavicle and the extent of damage to the acromioclavicular (AC) and coracoclavicular (CC) ligaments, among other factors [2,3]. While the injury often results from a direct blow to the shoulder, less commonly, a fall onto an outstretched hand or elbow can transfer indirect force to the shoulder, causing dislocation [4].

The Rockwood classification is the most widely used system for classifying ACJ injuries. This system divides the injury into six types based on the degree and direction of displacement of the clavicle with respect to the acromion [5]. Type I and II injuries are considered incomplete and are usually managed conservatively. However, Type IV, V, and VI injuries are considered extremely unstable and are treated surgically [6]. The management of Type III injuries is still a topic of debate. Some patients have done well with conservative management, while others have faced long-lasting pain and ongoing issues with stability [7]. To address these issues, surgical treatment for Type III injuries has been proposed as a means to restore ACJ structure and improve functional outcomes [8].

Clavicle hook plates, which are pre-contoured and available in various sizes to match individual anatomical structures, have been developed to treat acute ACJ dislocations. These plates are secured to the lateral one-third of the clavicle using screws and posteriorly beneath the acromion process, thereby maintaining the reduction of the joint [9]. This approach has shown effectiveness in managing Rockwood Type III and V dislocations, offering the advantage of preserving the ACJ while reducing dislocation [3]. Despite its success in treating ACJ injuries, the use of hook plates carries certain complications, such as compromise of the subacromial space, potential rotator cuff injuries, and the risk of acromial stress fractures due to the hook [10]. Therefore, assessing shoulder function following the reduction and fixation of a displaced ACJ is crucial for successful outcomes in managing ACJ dislocations [9].

## Materials and methods

This retrospective case series was done in the orthopedic department of Sheikh Zayed Medical College and Hospital, Rahim Yar Khan, Pakistan, over one year, from January 20, 2021, to January 19, 2022. The functional outcome was evaluated using the Constant Murley Score (CMS) on the day before plate removal, three months after plate removal, and six months following surgery. The CMS is presented in Table 1.

**Table 1:** Constant Murley Score

Parameter	Sub-parameter	Description	Points
Pain			0 - 15
	No pain	No pain at all	15
	Mild pain	Occasional, tolerable pain	10 - 14
	Moderate pain	Pain that affects daily activities	5 - 9
	Severe pain	Constant, severe pain	0 - 4
Activities of daily living (ADL)			0 - 20
	Sleep	Can sleep on the affected side	2
	Work and hobbies	Able to perform usual work/hobbies	Up to 4
	Positioning arm		0 - 10
	- Comb hair	Position of the arm that allows this activity	2
	- Wash the back	Position of arm for washing the back	2
	- Eat	Ablility to bring the hand to the mouth	2
	- Jacket	Ability to put on a jacket	2
	- Tuck in the shirt	Ability to reach back waistline	2
Range of motion (ROM)			0 - 40
	Forward elevation	0°–150°+	0 - 10
	Abduction	0°–150°+	0 - 10
	External rotation	Measure of outward shoulder rotation	0 - 10
	Internal rotation	Measure of inward shoulder rotation	0 - 10
Strength		Abduction force, measured in pounds or kilograms	0 - 25
		Measured at 90° abduction	

Inclusion and exclusion criteria

Patients included were those with unilateral closed ACJ dislocations classified as Rockwood Types III and V, with a history of trauma, aged between 18 and 60 years, regardless of gender, and presenting with injuries less than two weeks old. Patients with Rockwood Types I and II injuries, open wounds, multiple traumas, nerve or vascular damage, and fractures of the contralateral clavicle or humerus were excluded.

Eligible patients were identified through medical records and clinic documentation. Initial management was carried out in the emergency department according to the Advanced Trauma Life Support (ATLS) protocol. A thorough local examination of the affected shoulder was conducted, and patients received intravenous analgesics for pain relief. The injured limb was immobilized using a Lancaster sling. Multiple radiographic views were utilized to confirm ACJ dislocation and understand the pathoanatomy. These included the anteroposterior (AP) view to evaluate the degree of displacement, the axillary view to assess medial or lateral displacement, the Zanca view to provide a clearer visualization of the ACJ, and the scapular Y (lateral) view to evaluate alignment. Additionally, a weighted (stress) view was considered to observe any dynamic instability of the ACJ under load and to differentiate between Type III and Type V. Patients were informed about the risks and benefits of the surgical treatment, and informed consent was obtained.

Operative technique

Patients were positioned in a beach chair position to ensure better access and visualization of the ACJ during surgery. After adequate anesthesia, the affected limb was prepped and draped following standard aseptic protocols. A transverse incision was made along the posterior aspect of the clavicle to expose the ACJ and surrounding structures.

During dissection, careful handling of the soft tissues was prioritized to minimize trauma. The deltoid and trapezius fasciae were carefully elevated to access the AC joint while preserving the integrity of surrounding tissues. This meticulous approach aimed to reduce postoperative complications and facilitate better soft tissue healing. Inspection of the joint confirmed ACJ dislocation, and attention was given to any observable ligament damage around the joint.

Reduction of the ACJ was achieved and temporarily maintained using K-wires for stabilization. A 3.5 mm clavicle hook plate was then applied for final fixation, securing the joint in the reduced position. Damaged ligaments, though not formally repaired in this procedure, were assessed for stability and alignment to ensure optimal positioning of the joint and plate. Preservation of the diarthrodial properties of the AC joint was also considered during plate placement to support functional outcomes.

Postoperatively, the limb was immobilized in a polysling to maintain stability and comfort. Patients received intravenous antibiotics and analgesics for 24 hours to manage infection risk and alleviate discomfort. Anteroposterior and scapular Y (lateral) shoulder radiographs were taken on the first postoperative day to verify the proper positioning of the joint and hook plate. After wound inspection and dressing change, patients were discharged on the second postoperative day with clear instructions for follow-up, including suture removal and postoperative care.

Follow-up assessments were conducted by reviewing clinic records at six, 12, and 24 weeks post surgery to ensure adherence to rehabilitation protocols. Routine removal of the hook plate was performed 24 weeks postoperatively to prevent potential complications such as acromion irritation or rotator cuff impingement. Post-implant removal, patients continued with rehabilitation, and their progress was evaluated through medical and clinic records at two weeks, three months after plate removal, and one year post surgery. During each follow-up, radiographs of the operated shoulder were obtained to confirm the maintenance of ACJ reduction and assess the CC distance, and no stress views were taken during these follow-up assessments.

## Results

This study presents the outcomes of 40 cases of ACJ stabilization using a hook plate for Rockwood Types III and V dislocations. The results were assessed both clinically, utilizing the CMS, and radiologically through anteroposterior shoulder X-rays taken at 24 weeks post surgery and eight weeks following plate removal. The mean age of the patients was 35.7 ± 9.85 years, with a male predominance (28 males, 70%) compared to females (12 females, 30%). Right-handedness was prevalent in 77.5% of the cases, involving 31 patients. The average injury-to-surgery interval (ISI) was 2.3 days. Among the 40 cases, 30 (75%) were classified as Rockwood Type III and 10 (25%) as Type V. This is presented in Table 2. 

**Table 2:** Demographic data of the patients

Variable	Frequency (n)			Percentage (%)
Gender				
Male		28		70.0
Female		12		30.0
Affected side				
Right			31	77.5
Left			9	22.5
Rockwood type				
Type III			30	75.0
Type V			10	25.0
Fracture cause				
Fall from height			7	17.5
Road travel accident (RTA)			30	75.0
Sports injury			3	7.5
Total			40	100.0
Mean injury-to-surgery interval (days)			2.13±.79	

At 24 weeks post plating, the mean pain component of the CMS was 8.60 ± 1.52, which improved to 9.65 ± 1.42 at eight weeks after plate removal, indicating a reduction in pain. The CMS assesses pain on a scale where higher scores represent less pain and better shoulder function, contrasting with the VAS, where higher scores indicate worsening pain.

The mean strength score in the CMS was 20.95 ± 1.01 at 24 weeks, increasing to 24.20 ± 0.61 eight weeks after plate removal, reflecting an improvement in strength. Similarly, the mean range of motion (ROM) score within the CMS was 26.05 ± 1.15 at 24 weeks and improved to 32.83 ± 2.14 eight weeks after plate removal, indicating enhanced mobility.

Regarding activity scores, the mean was 16.15 ± 0.83 at 24 weeks after plating, rising to 20.83 ± 1.06 eight weeks after the removal of the plate. For the CMS, 24 weeks post surgery, the mean score was 72.25 ± 3.95, with 16 patients (40%) scoring 70 and eight patients (20%) scoring 72, indicating a satisfactory outcome. Eight weeks after plate removal, the mean CMS increased to 90.45 ± 1.95, with 24 patients (60%) scoring 90 and five patients (12.5%) scoring 92, demonstrating excellent functional outcomes (Table 3).

**Table 3:** Patients' functional outcomes

	24^th^ week after plating	8^th^ week after plate removal
Pain score (Mean±SD)	8.60±1.52	9.65±1.42
Activity score (Mean±SD)	16.15±.83	20.83±1.06
Range of motion (ROM) score (Mean±SD)	26.05±1.15	32.83±2.14
Strength score (Mean±SD)	20.95±1.01	24.20±0.61
Constant score (Mean±SD)	72.25± 3.95 (Satisfactory)	90.45± 1.95 (Excellent)

## Discussion

Acromioclavicular joint dislocation is a common shoulder injury that greatly affects upper limb function and daily activities. Demographic studies have shown a higher number of ACJ injuries among men, especially in young individuals [1, 3]. While various surgical techniques have been used to maintain ACJ reduction, implant loosening or breakage due to stress concentration and non-dynamic fixation have been documented [11]. In comparison, the clavicle hook plate functions as a dynamic fixation device, providing fixation as the hook of the plate is passed under the acromion and the plate is secured on top of the clavicle with screws. This mechanism maintains the clavicle's position and CC distance, resulting in more favorable outcomes. Studies have indicated that hook plate fixation of the ACJ replicates the normal biomechanics of the ACJ, making it one of the most preferred treatments for ACJ dislocation [12].

This retrospective study evaluated the outcomes of 40 cases of ACJ stabilization using a hook plate for Rockwood Types III and V dislocations. In our study, the mean age for the patients was 35.7 ± 9.85 years, with a male predominance (28 men, 70%) compared to women (12 women, 30%). Right-handedness was predominant, accounting for 77.5% of the cases, involving 31 patients. The average ISI was 2.3 days. Among the cases, 30 (75.0%) were classified as Rockwood Type III and 10 (25%), as Type V. These findings are consistent with other studies in the literature [1, 3, 13, 14].

The mean CMS at eight weeks following plate removal in our study was 90.45 ± 1.95. Similar findings were reported by Kumar and Sharma, who treated 33 patients with ACJ dislocation using clavicular hook plates. Their outcomes were evaluated using a continuous shoulder score, which revealed an average score of 91.3 at the latest follow-up [14]. Another prospective study involving 20 patients treated with a hook plate for ACJ dislocation reported an average CMS of 92.9 at the final follow-up after plate removal at three months [15]. Additionally, Hemmann et al. conducted a study in 2020 on 99 patients with ACJ dislocation managed with a hook plate, finding excellent outcomes at the final follow-up [16].

Limitations

This study has several limitations that should be considered when interpreting the results. First, the sample size was relatively small, with only 40 patients included, which may limit the generalizability of the findings to a larger population. Additionally, the study was conducted as a retrospective case series, relying on medical and clinic records for data collection. This approach may introduce recall bias and limit the ability to control for confounding factors.

Another limitation is the lack of a control group or comparison with other treatment modalities, such as alternative surgical techniques or conservative management, which restricts the assessment of the clavicular hook plate's effectiveness relative to other methods. The follow-up period was limited to one year, which may not capture long-term outcomes or potential late complications associated with hook plate fixation, such as osteoarthritis or implant-related issues.

## Conclusions

This study demonstrates that clavicular hook plate fixation is an excellent treatment option for ACJ dislocations of Rockwood Type III and Type V. The procedure yields favorable functional outcomes when considering pain, regular activity, range of motion, and shoulder strength. Twenty-four weeks post surgery, the mean pain, strength, ROM, and activity scores showed significant improvement, indicating a satisfactory recovery. Further improvements in these parameters were observed eight weeks after plate removal, with the CMS reaching an average of 90.45 ± 1.95, demonstrating an excellent functional outcome.

Our findings align with previous studies, suggesting that clavicular hook plate fixation effectively maintains the ACJ's stability while preserving its biomechanics. Moreover, the small number of postoperative complications such as implant loosening or breakage highlights the procedure's reliability. Thus, clavicular hook plate stabilization can be considered a preferred method for treating acute ACJ dislocations, providing both radiographic and functional benefits.
